# Influence of Stereochemistry on the Monolayer Characteristics of *N*-alkanoyl-Substituted Threonine and Serine Amphiphiles at the Air–Water Interface

**DOI:** 10.1021/acs.langmuir.1c01108

**Published:** 2021-07-21

**Authors:** G. Brezesinski, F. Strati, R. Rudert, D. Vollhardt

**Affiliations:** † Institute for Applied Dermatopharmacy, Martin Luther University Halle-Wittenberg, Weinbergweg 23, D-06120 Halle, Germany; ‡ Section of Chemical Information Systems, University of Ulm, D-89081 Ulm, Germany; § Max-Planck Institute for Polymer Research, Ackermannweg 10, D-55128 Mainz, Germany

## Abstract

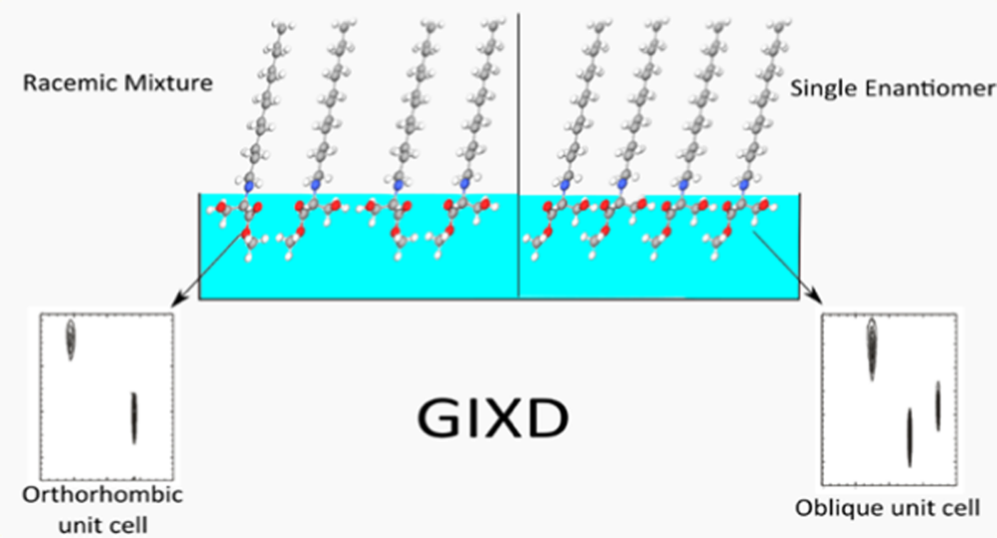

Thermodynamic and structural properties of the *N*-alkanoyl-substituted α-amino acids threonine and serine, differing only by one CH_3_ group in the head group, are determined and compared. Detailed characterization of the influence of stereochemistry proves that all enantiomers form an oblique monolayer lattice structure whereas the corresponding racemates build orthorhombic lattice structures due to dominating heterochiral interactions, except *N*-C16-dl-serine-ME as first example of dominating homochiral interactions in a racemic mixture of *N*-alkanoyl-substituted α-amino acids. In all cases, the liquid expanded–liquid condensed (LE/LC) transition pressure of the racemic mixtures is above that of the corresponding enantiomers. Phase diagrams are proposed. Using the program Hardpack to predict tilt angles and cross-sectional area of the alkyl chains shows reasonable agreement with the experimental grazing incidence X-ray diffraction (GIXD) data.

## Introduction

Monolayers of amino acid-type amphiphiles are frequently used as model systems to understand complicated physical–chemical factors responsible for the mechanisms effective in biological membranes. A methodology to combine nanotechnology and these organization processes was recently proposed as a novel concept of nanoarchitectonics, which can fabricate functional materials with nanolevel units. For instance, according to the chiral modulation, the chiral selectivity of amino acids can be desirably tuned only with simple macroscopic mechanical compression of the receptor film.^1^

Based on the good biodegradability and low toxicity of amino acid-type amphiphiles, their monolayers have been of relevance to obtain information on (bio)sensing mechanisms, drug delivery processes, two-dimensional (2D) chiral organization and recognition, etc. and for the development of sensors, corrosion inhibitors, anti-biofouling layers, etc.^2^

Langmuir monolayers of *N*-alkanoyl-substituted α-amino acids have been traditional challenges for experimental studies on mesoscopic and microscopic level using surface pressure–molecular area (π–A) isotherms,^3−9^ Brewster angle microscopy (BAM),^10−14^ grazing incidence X-ray diffraction (GIXD), and infrared reflection-absorption spectroscopy.^15−18^

Langmuir monolayers of different amino acid derivatives were recently studied on several subphases.^19^ Some years ago, the influence of cis/trans stereochemistry on the condensed-phase and monolayer structure of chiral cyclobutane β-amino acid-based amphiphiles was demonstrated.^20^

Generally, the main monolayer characteristics of *N*-alkanoyl-substituted α-amino acid amphiphiles show substantial differences to the usual amphiphilic monolayers. Furthermore, monolayers of different *N*-alkanoyl-substituted α-amino acid amphiphiles have been used as easy to process model systems to demonstrate the influence of their head group structure on the main monolayer characteristics. A large variety of condensed-phase domain shapes obtained from different *N*-alkanoyl-substituted α-amino acid molecules were observed.^11−14^ However, so far more efforts to draw some general conclusions about the relationship of domain topography and the molecular structure are necessary.^21^

In a recent study, detailed characterization of the monolayers of two *N*-alkanoyl-substituted threonine amphiphiles in their chiral and racemic states on mesoscopic and molecular scales indicated substantial differences to usual amphiphilic monolayers.^22^ Generally, surface pressure–molecular area (π–A) measurements of the enantiomeric and racemic forms indicated that all compression curves are located above the corresponding decompression curves, and the transition pressures at a fixed temperature of the racemic forms are always below those of the enantiomeric forms.

It is interesting to note that threonine, having two chiral centers, can exist in four possible stereoisomers. In addition, the usual stereoisomer threonine with the configuration (2S, 3R), the stereoisomer (2S, 3S), called *L*-*allo*-threonine, is also present in nature. The effect of the second chiral center of the diastereomeric *N*-alkanoyl-*allo*-threonine on the main monolayer characteristics has been recently studied.^23^ Comparison of the special thermodynamic and structural features of the two stereomers *N*-alkanoyl–threonine and *N*-alkanoyl-*allo*-threonine, in both enantiomeric and racemic monolayers, has shown that the diastereomeric head group structure strongly affects the specific characteristics.

Generally, there are possibilities to describe the properties of the molecular structures of the condensed-phase domains at various levels of detail (tetrahedral model, coarse grained, atomistic) and to calculate their intermolecular energy profile. Effective pair potential (EPP) theory of groups attached to the chiral center was applied to monolayers of amino acid amphiphiles (*N*-palmitoylaspartic acid, *N*-stearoylserine methylester, *N*-palmitoyl-*allo*-threonine methylester, *N*-stearoyl-*allo*-threonine methylester) to predict the handedness of domains composed of these molecules.^24,25^

In a recent study, the thermodynamic and structural parameters of *N*-alkanoyl-substituted alanine monolayers were calculated on the basis of the quantum chemical semiempirical PM3 method.^26^

The program Hardpack was used to predict possible two-dimensional packing arrangements. For comparison with the experimental GIXD data, the two-dimensional lattice parameters and characteristic features of the enantiomeric and racemic diastereomeric stearoyl-threonine monolayers were calculated and are in reasonable agreement with the experimental GIXD data.^23^

This raises the question of how specific structure components of the head group influence the monolayer characteristics. Therefore, the main monolayer characteristics of the two *N*-alkanoyl-substituted α-amino acid amphiphiles threonine and serine differing only by the CH_3_ group in the head group are determined and compared. Furthermore, this study provides information on how the introduction of a methylester into the head group of both amphiphiles affects their characteristic features. Finally, the present study aims at a detailed characterization of the influence of stereochemistry on the thermodynamic and structural features of the corresponding monolayers.

For each head group type, quantum chemical calculations of the molecular structure were performed to theoretically predict the two-dimensional packing and to compare this with the experimental findings.

## Experimental Section

**Figure 1:**
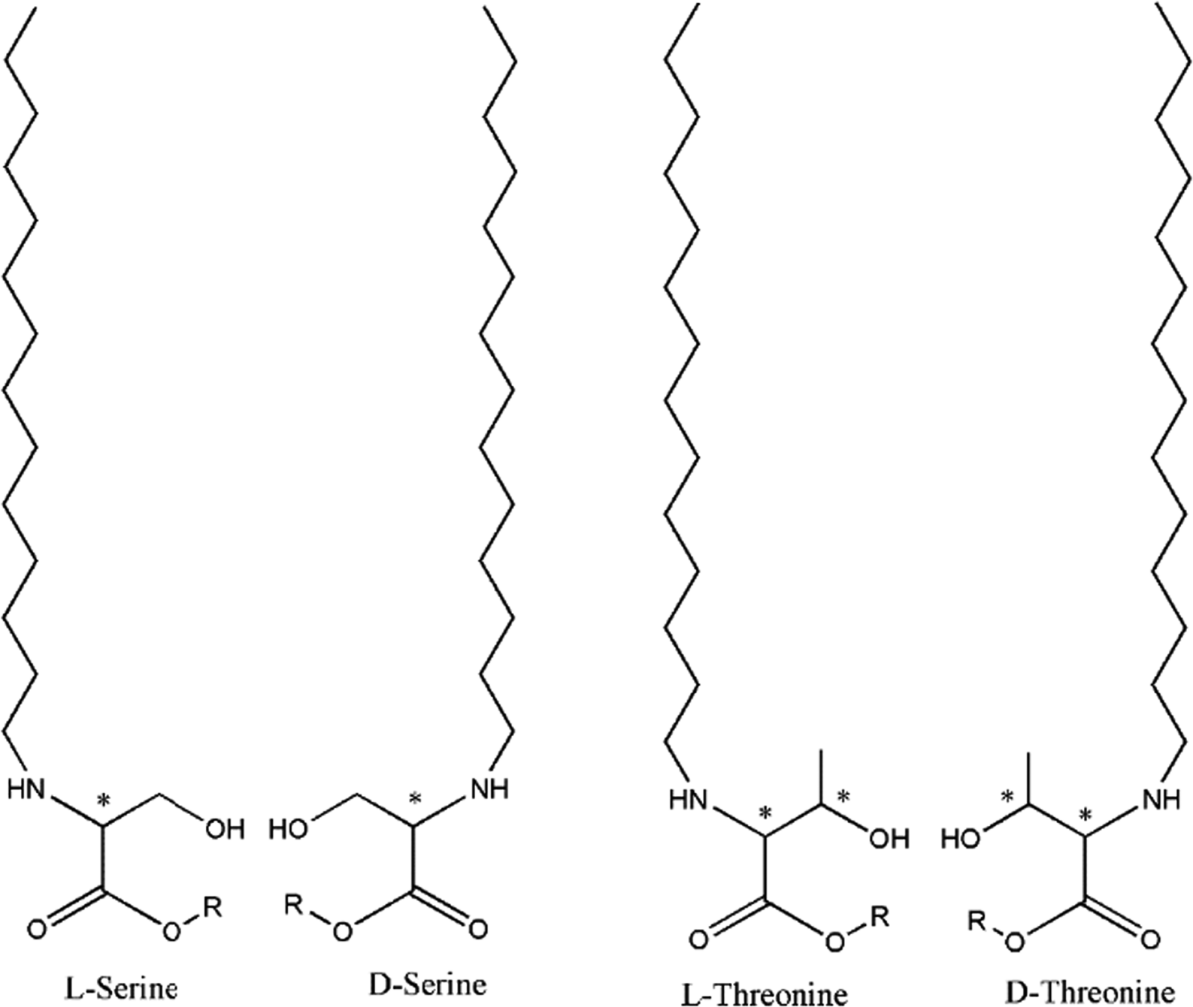
Chemical structures of the investigated *N*-alkanoyl-substituted serine and threonine amphiphiles with R = H for the serine and threonine amphiphiles and R = CH_3_ for the corresponding methylesters. The chiral centers are marked with an asterisk. Threonine has two chiral centers and therefore four possible stereoisomers with the following configurations: (2S, 3R), (2R, 3S), (2S, 3S), and (2R, 3R).

The enantiomeric *N*-stearoyl-substituted threonine methylester amphiphiles *N*-C18-d-threonine-ME and *N*-C18-l-threonine-ME and the enantiomeric *N*-palmitoyl-substituted serine-methylester amphiphiles *N*-C16-d-serine-ME and *N*-C16-l-serine-ME (Figure 1) were synthesized by condensation of chiral threonine- or serine-methylesters and stearoyl, respectively, palmitoyl chloride in chloroform and aqueous potassium carbonate.^27^ The *N*-stearoyl-, respectively, *N*-palmitoyl-substituted threonine serine amphiphiles *N*-C18-d-threonine, *N*-C18-l-threonine, *N*-C16-d-serine, and *N*-C16-l-serine (Figure 1) were obtained by hydrolyzing stearoyl-threonine-, respectively, palmitoyl-serine-methylesters with sodium hydroxide in aqueous dioxane.^27^ In all cases, the obtained reaction products were purified by repeated crystallization in methanol. The chemical and chiral purities (99%) of the final products were confirmed by elemental analysis and high-performance liquid chromatography (HPLC).

The surface pressure–molecular area (π–A) isotherms were recorded with a self-made, computer-interfaced film balance using the Wilhelmy method with a roughened glass plate.^28^ The *N*-alkanoyl-threonine/serine amphiphiles, dissolved in a heptane/ethanol (9:1) (Merck p.a. grade) mixture, were spread on pH 3 water. The used water has a specific resistance of 18.2 MΩ·cm, purified by a Millipore desktop system, and the pH was adjusted by HCl. Compression and decompression curves were measured at a compression rate of ≤10 Å^2^/(molecule·min) with an accuracy of the surface tension of ±0.1 mN/m and the molecular area of ±0.5 Å^2^.

The grazing incidence X-ray diffraction measurements were performed at the liquid-surface diffractometer (BW1, HASYLAB, DESY, Hamburg, Germany).^29^ The thermostated Langmuir film balance was positioned in an air-tight container with a Kapton (polyimide film) window under a helium atmosphere. During the experiment, a monochromatic X-ray beam (λ = 1.304 Å) strikes the water surface at a grazing incidence angle α_*i*_ = 0.85α_c_ (where α_c_ = 0.13° is the critical angle for total reflection of the X-ray beam at the water surface) illuminating approximately 2 × 50 mm^2^ monolayer surface. A slow lateral movement of the trough is used to avoid sample damage by the strong X-ray beam. The diffracted signal was measured by a linear position-sensitive MYTHEN detector system (PSI, Villigen, Switzerland). The in-plane *Q*_*xy*_ component of the scattering vector was scanned by rotation of the detector around the sample in the *x*–*y* plane, and the out-of-plane *Q*_*z*_ component of the scattering vector was obtained using the vertical strips of the MYTHEN between 0.0 and 0.75 Å^–1^. The analysis of the diffraction patterns generates the lattice structures. The Bragg peaks are obtained by integration of the scattering intensity (corrected for polarization, effective area, and Lorentz factor) over a certain *Q*_*z*_ window and the Bragg rods by the integration of the scattering intensity over a certain *Q*_*xy*_ window. The unit cell dimensions (lattice parameters *a*, *b*, *c*, in-plane area *A*_*xy*_, cross-sectional area *A*_0_, tilt angle *t*) are calculated from the peak positions. More details can be found in the literature.^30−33^

For the theoretical prediction of the two-dimensional packing, the configuration of the single molecules was optimized by quantum chemical calculations with the program Gaussian 09.^34^ The molecular packing was calculated by a Monte Carlo algorithm using the program Hardpack.^35^ This program tries to find the global minimum of the energy of the two-dimensional packing arrangements of molecules. The energy is calculated from van der Waals and electrostatic interactions between different molecules and rotatable groups. The bond length and bond angles of the molecules are fixed. Selected groups are allowed to rotate. Theoretical point charges at the atom positions are used. The two-dimensional space group p1 was used for the packing of the enantiomers, whereas the rectangular space group pg was applied for the packing of the racemates. The molecular structures were optimized by quantum chemical calculations with the program Gaussian 09^34^ using the B3LYP method and the 6-31G basis set. For each packing type, a few hundred molecular packing arrangements with varying parameters, including six to seven internal rotations, were computed. Packing arrangements with crossed chains were excluded. Polarization and thermal motion were ignored. The parameters for the van der Waals interactions were taken from the DREIDING force field^36^ and the point charges from the Gaussian 09 calculations.

## Results and Discussion

In recent papers,^22,23^ we have investigated the influence of stereoisomery on the thermodynamic characteristics of *N*-alkanoyl-substituted threonine and *allo*-threonine monolayers. In the present manuscript, we study the effect of small chemical changes in the head group structure using *N*-alkanoyl-substituted serine and threonine, which can be considered as 3-methyl-serine amphiphiles and compare them with the corresponding methylesters.

First, information about the influence of stereochemistry on the thermodynamic characteristics is obtained by comparison of the experimental π–A curves of the enantiomeric and racemic monolayers measured at different temperatures.

**Figure 2:**
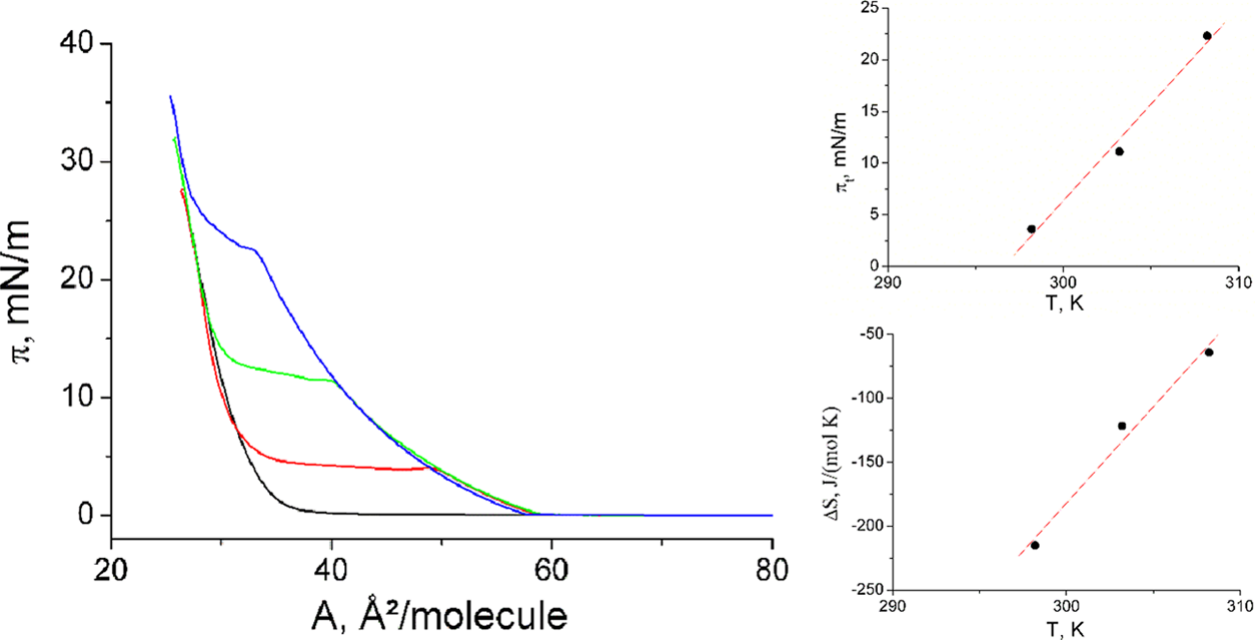
Left: π–A curves of *N*-C16-dl-serine at 20 °C (black), 25 °C (red), 30 °C (green), and 35 °C (blue) on pH 3 water. Right top: Temperature dependence of the main phase-transition pressure π_t_ at the liquid expanded–liquid condensed (LE/LC) phase transition. Right bottom: Temperature dependence of the entropy change Δ*S* at the LE/LC phase transition.

In Figure 2, the pressure–area isotherms of *N*-C16-substituted serine monolayers are presented together with the thermodynamic data, which can be determined only for the racemate. The isotherms exhibit a behavior similar to that reported for the corresponding threonine.^22^

The compression isotherms of the racemic *N*-C16-serine on pH 3 water exhibit only a marginal overcompression to induce the nucleation of the condensed phase. This is in contrast to the corresponding threonines.^22^ Obviously, the energy barrier to start the nucleation in threonine monolayers is increased by steric hindrance due to the introduced methyl group.

Figure 2 (right top) presents the π_t_–*T* relationship. The temperature dependence of the phase-transition pressure (π_t_), corrected by the observed overcompression, allows the determination of the *T*_0_ values, which are the lowest temperature at which the liquid expanded (LE) phase can be observed. Below *T*_0_, the monolayer transforms directly from the gas-analogous state into a condensed one (resublimation). The slope dπ_t_/d*T* of the linear fit to the experimental data amounts to 1.87 mN/(m·K), and is therefore slightly larger compared to that of the *N*-C16-dl-threonine (1.63 mN/(m·K)). The linearly fitted curves reach zero transition pressure at *T*_0_ = 296.6 K (23.4 °C) very different from 274.3 K (1.1 °C) obtained for *N*-C16-dl-threonine.

Access to the transition entropy (Δ*S*), presented in Figure 2 (right bottom), is obtained by the temperature dependence of the phase-transition pressure (π_t_) and the area change (ΔA) during the first-order LE/LC transition using the two-dimensional Clapeyron equation, Δ*S* = ΔA × dπ_t_/d*T*. According to the exothermic nature of the main phase transition at compression of amphiphilic monolayers, negative Δ*S* values are obtained. The absolute Δ*S* values increase with decreasing temperature, indicating the increase of the condensed-phase ordering at lower temperatures. The linear fit and extrapolation to zero Δ*S* yields the critical temperature *T*_c_, above which the monolayer cannot be compressed into the condensed state. The observed critical temperature *T*_c_ = 312.1 K (38.9 °C) is also much larger compared to that of *N*-C16-dl-threonine (287.1 K). The temperature range for the existence of the LE phase is for the racemic form with 15.5 K, only a little larger than that of *N*-C16-dl-threonine (12.8 K) but distinctly (∼24 K) shifted to higher temperatures.

**Figure 3:**
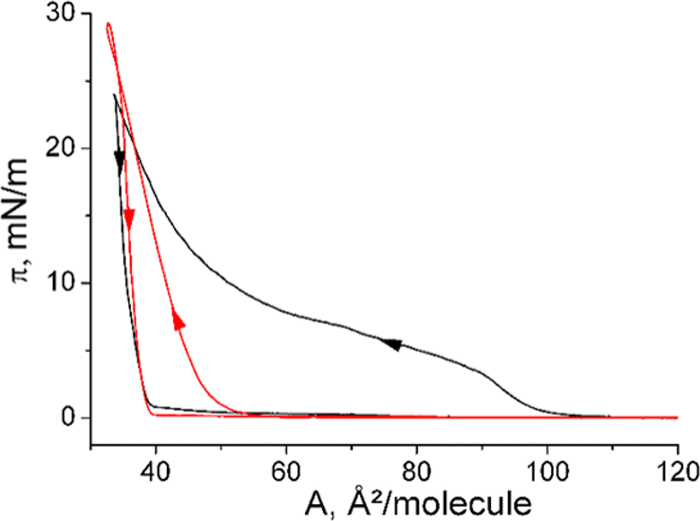
π–A curves of *N*-C16-l-serine during compression and expansion (indicated by the arrows) at 20 °C on pH 3 water. The first compression–expansion cycle (black) demonstrates the first-order LE/LC transition only during compression, whereas the second compression–expansion cycle (red) does not show this transition.

The corresponding enantiomer *N*-C16-l-serine behaves very strange. The comparison between the compression and expansion curves documents a strong nonequilibrium behavior. Therefore, we did not attempt to extract thermodynamic data. Representative compression and expansion curves obtained at 20 °C are presented in Figure 3.

The first-order LE/LC transition is only during the first compression present. The first expansion curve is typical for the transition of the LC phase directly into the gas-analogous phase (sublimation). Even expansion and 15 min relaxation time do not lead to the complete reappearance of the LE phase. The second compression curve does not exhibit the LE/LC transition plateau but is only slightly shifted to slightly larger areas. Obviously, a much longer waiting time at zero pressure (large molecular areas) is needed for the formation of the liquid-like LE phase.

**Figure 4:**
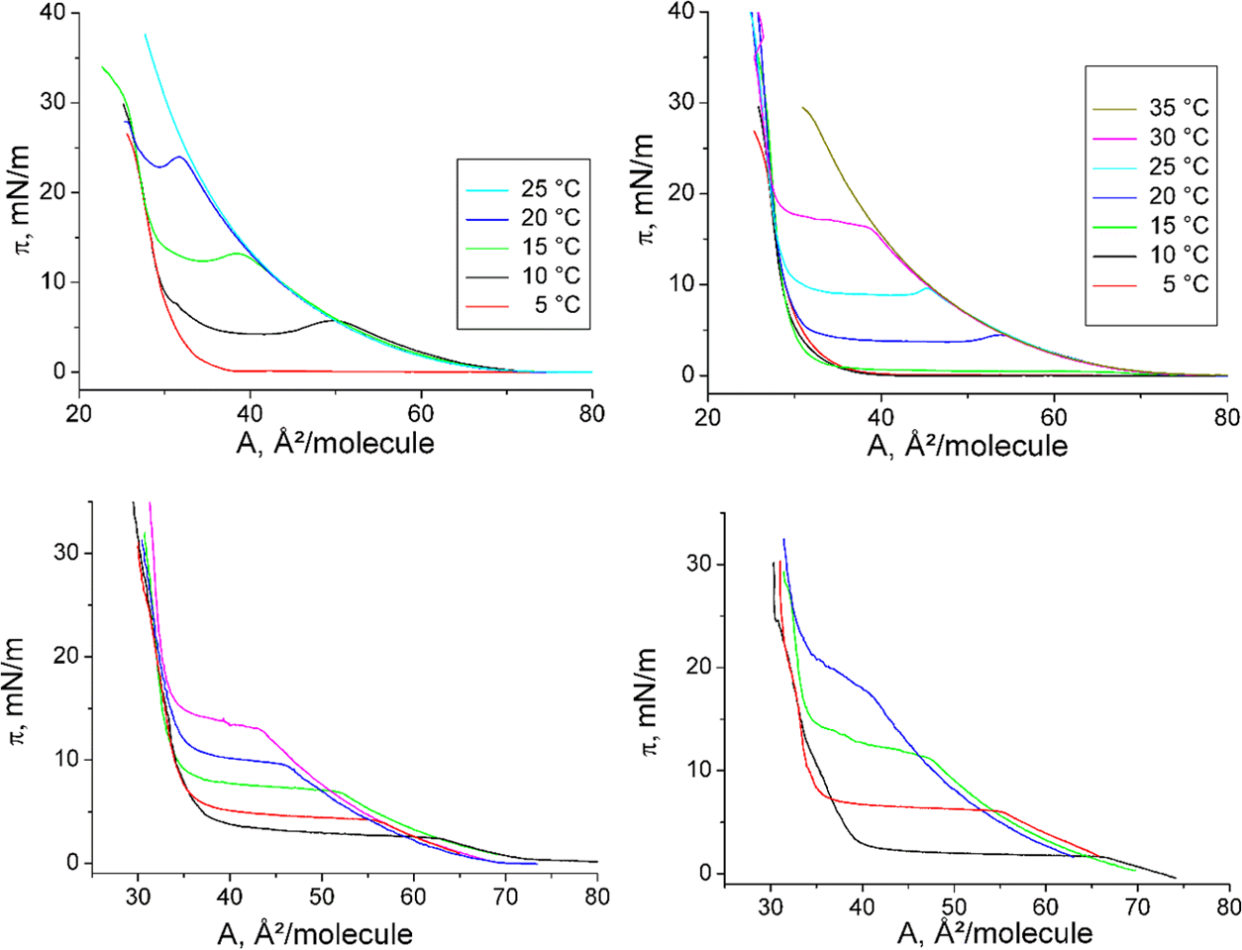
Top: π–A curves of *N*-C16-dl-serine-ME (left) and *N*-C16-l-serine-ME (right) monolayers at different temperatures (indicated) on pH 3 water. Bottom: Expansion π–A curves of *N*-C18-dl-threonine-ME (left) and *N*-C18-l-threonine-ME (right) monolayers at different temperatures (racemate: 12 °C (black), 14 °C (red), 16 °C (green), 18 °C (blue), and 20 °C (magenta); enantiomer: 16 °C (black), 20 °C (red), 24 °C (green), and 28 °C (blue)) on water.

The compression isotherms of both enantiomeric and racemic *N*-C16-serine-ME indicate a slight overcompression to induce the nucleation of the condensed phase (Figure 4, top).

The π_t_–*T* relationship of the *N*-C16-substituted serine-methylester monolayers on pH 3 water is presented in Figure S1 (left). The slope dπ_t_/d*T* of the linear fit amounts to 1.27 mN/(m·K) for *N*-C16-l-serine-ME and 1.80 mN/(m·K) for *N*-C16-dl-serine-ME. The latter value is similar to that of the corresponding *N*-C16-dl-serine (1.87 mN/(m·K)). The *T*_0_ values are 290.8 K (17.6 °C) for *N*-C16-l-serine-ME and 281.3 K (8.1 °C) for *N*-C16-dl-serine-ME. The Δ*T*_0_ of 9.5 K between the enantiomeric (l) and the racemic (dl) forms is quite large. The introduction of the methylester shifts the *T*_0_ value of the racemate to much lower temperatures.

The temperature dependence of the transition entropy (Δ*S*) is presented in Figure S1 (right). For the racemate, the *T*_c_ value is also shifted by ∼15 K to lower temperatures compared to the corresponding serine monolayer. The Δ*T*_c_ difference between the enantiomeric and the racemic forms is with 14.2 K even larger than the Δ*T*_0_ difference. The characteristic temperatures *T*_0_ (π_t_ = 0) and *T*_c_ (Δ*S* = 0) are listed in Table 1.

**Table 1:** Characteristic Temperatures *T*_0_ (π_t_ = 0) and *T*_c_ (Δ*S* = 0) of the Studied Monolayers

	*T*_0_ (K) enantiomer	*T*_0_ (K) racemate	*T*_c_ (K) enantiomer	*T*_c_ (K) racemate	Δ*T*_0_	Δ*T*_c_
*N*-C16-serine		296.6		312.1		
*N*-C16-serine-ME	290.8	281.3	310.7	296.5	9.5	14.2
*N*-C16-threonine	275.2	274.3	291.1	287.1	0.9	4.0
*N*-C18-threonine	292.5	289.6	304.2	302.4	2.9	1.8
*N*-C18- threonine-ME	288.2	283.9	304.8	297.3	4.3	7.5

The temperature range for the existence of the LE phase is for the racemic form with ∼15 K clearly smaller than for the enantiomeric form (∼20 K), but very similar compared to the corresponding serine.

The expansion π–A curves of the enantiomeric and racemic *N*-C18-substituted threonine methylester monolayers have been measured at different temperatures (Figure 4, bottom). Since no charge is involved, the experiments have been performed on a water subphase. The compression isotherms of both enantiomeric and racemic *N*-C18-threonine-ME are connected with an overcompression to induce the nucleation of the condensed phase (representative example is shown in Figure S2). The temperature dependence of the phase-transition pressure (π_t_) has therefore been determined from the expansion curves which can be regarded as equilibrium isotherms (Figure 4, bottom) as described for the corresponding threonine monolayers.^19^ The slope dπ_t_/d*T* of the linear fit to the experimental data amounts to 1.31 mN/(m·K) for the enantiomer and 1.34 mN/(m·K) for the racemate (Figure S3). The linearly fitted curves reach zero transition pressure at *T*_0_ = 288.2 K (15.0 °C) for *N*-C18-l-threonine-ME and 283.9 K (10.7 °C) for *N*-C18-dl-threonine-ME. The Δ*T*_0_ = 4.3 K between the enantiomeric (l) and the racemic (dl) forms is much smaller than the one observed for the serine methylesters. This could be due to the larger chain length (C18–C16) with increased van der Waals interactions between the chains which might diminish the weak chirality influence. Compared with the *N*-C18-substituted threonine monolayers, the slope dπ_t_/d*T* is only similar (1.34–1.25 mN/(m·K)) for the racemates but very different (1.31–1.77 mN/(m·K)) for the corresponding enantiomers. However, the *T*_0_ and *T*_c_ values are not much influenced by the introduction of the methylester (see Table 1). The characteristic temperatures of *T*_0_ (π_t_ = 0) and *T*_c_ (Δ*S* = 0) are listed in Table 1. The Δ*T*_c_ difference between the enantiomeric and the racemic forms is with 7.5 K larger than the Δ*T*_0_ value, but again much smaller compared to the Δ*T*_c_ difference of the serine methylesters with the shorter chain. Compared to the corresponding *N*-C18-substituted threonine monolayers, the *T*_0_ and *T*_c_ are not considerably changed by the methylester (see Table 1).

The temperature range for the existence of the LE phase is for the racemic form with ∼13 K again smaller than for the enantiomeric form (∼17 K), but the difference is clearly smaller compared to *N*-C16-serine-ME. In the case of *N*-C18-substituted threonine monolayers, the LE phase exists over a temperature range of ∼12 K for the enantiomer and ∼13 K for the racemate. This shows that the introduction of the methylester influences this temperature range only for the enantiomer, while it is not changed for the racemic form.

GIXD studies provide information about the characteristic features of the lattice structure of condensed monolayer phases on the Angstrom scale. GIXD has been used to elucidate the influence of stereochemistry on the lattice structure of all amphiphiles investigated in this study. The GIXD measurements were performed at 10 °C and at different lateral pressures except for *N*-C16-l-serine for which only 2 mN/m was investigated as lateral pressure due to the monolayer instability.

**Figure 5:**
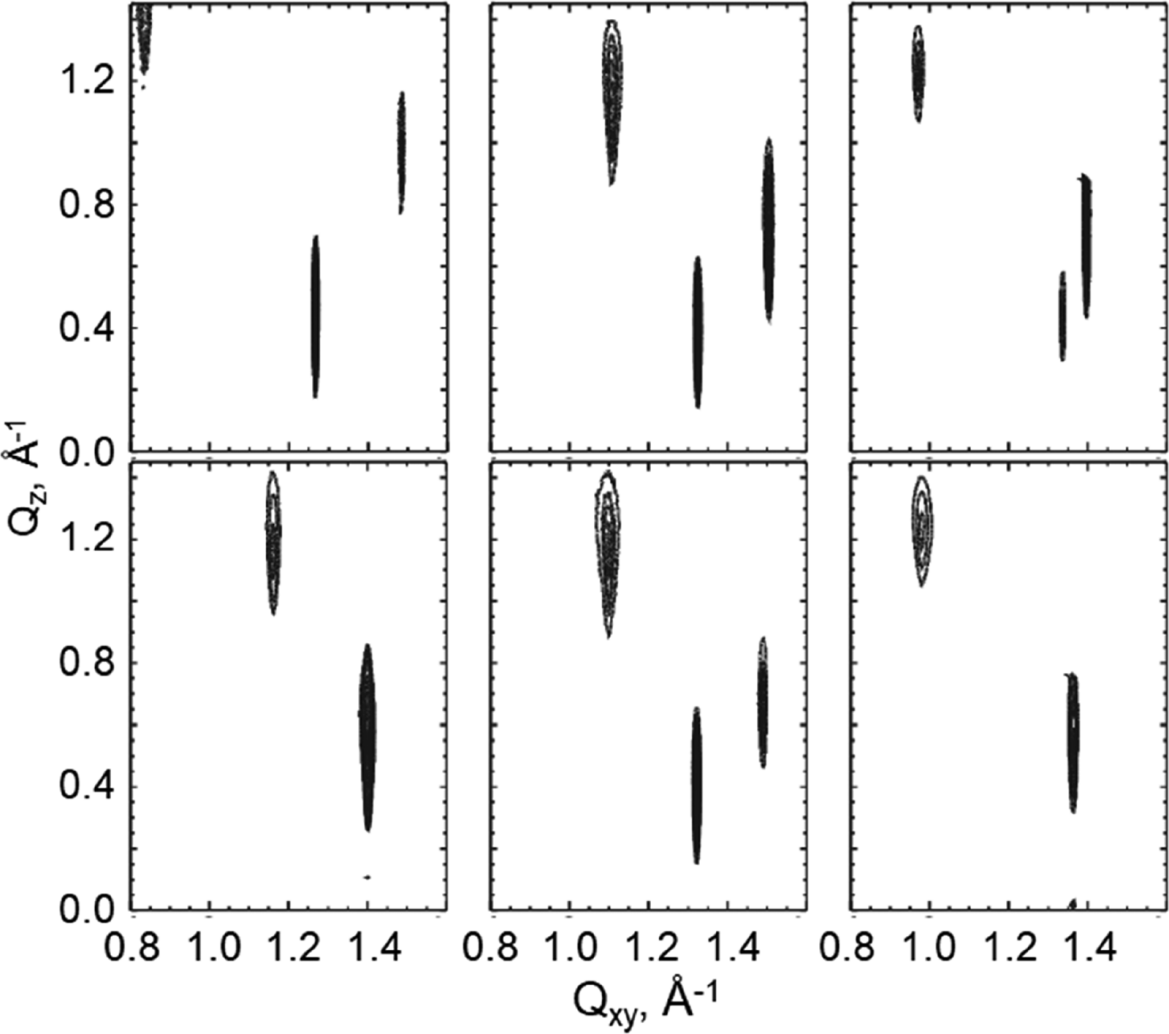
Contour plots of equal intensity vs the in-plane component *Q*_*xy*_ and the out-of-plane component *Q*_*z*_ of the scattering vector of enantiomeric (upper row) and racemic (lower row) *N*-C16-serine (left), *N*-C16-serine-ME (middle), and *N*-C18-threonine-ME (right) monolayers. The racemates form a NNN tilted orthorhombic structure (two out-of-plane diffraction peaks) except *N*-C16-dl-serine-ME, whereas all enantiomers exhibit an oblique unit cell (three diffraction peaks).

Figure 5 presents selected contour plots of equal intensity versus the in-plane component *Q*_*xy*_ and the out-of-plane component *Q*_*z*_ of the scattering vector for *N*-C16-dl-serine, *N*-C16-l-serine, *N*-C16-dl-serine-ME, *N*-C16-l-serine-ME, *N*-C18-dl-threonine-ME, and *N*-C18-l-threonine-ME. In the case of the enantiomers, all amphiphiles exhibit three Bragg peaks at all pressures along the isotherm. This shows that the enantiomers form an oblique lattice structure, as expected for chiral compounds. The Bragg peak positions, their full widths at half-maximum, and lattice parameters obtained at different surface pressures are listed in Supporting Information (SI) Tables S1–S3. For direct comparison, Table 2 presents selected lattice data for the enantiomeric and racemic monolayers under study.

**Table 2:** Top: Bragg Peak and Rod Positions and the Corresponding Full Widths at Half-Maximum of Racemic and Enantiomeric *N*-C16-Serine (2 mN/m), *N*-C16-Serine-ME (dl: 15 mN/m and l: 17 mN/m), and *N*-C18-Threonine-ME (15 mN/m) Monolayers^a^

compound	*Q_xy_* (Å^–1^)	*Q_z_* (Å^–1^)	*Q_xy_* (Å^–1^)	*Q_z_* (Å^–1^)	*Q_xy_* (Å^–1^)	*Q_z_* (Å^–1^)
*N*-C16-dl-serine			1.152	1.16	1.400	0.58
			0.024	0.31	0.009	0.29
*N*-C16-l-serine	0.834	1.33	1.267	0.41	1.485	0.92
	0.044	0.30	0.009	0.30	0.020	0.30
*N*-C16-dl-serine-ME	1.099	1.109	1.323	0.41	1.490	0.68
	0.040	0.3	0.009	0.28	0.017	0.29
*N*-C16-l-serine-ME	1.108	1.12	1.325	0.42	1.504	0.70
	0.034	0.30	0.008	0.28	0.013	0.29
*N*-C18-dl-threonine-ME			0.981	1.16	1.364	0.58
			0.045	0.27	0.014	0.28
*N*-C18-l-threonine-ME	0.979	1.15	1.340	0.49	1.402	0.66
	0.042	0.28	0.013	0.27	0.017	0.28

compound	*a*, *b*, *c* (Å)	α, β, γ (deg)	*d*	*t* (deg)	*A_xy_* (Å^2^)	*A*_0_ (Å^2^)
*N*-C16-dl-serine	4.924	131.4	0.2412	45.2	26.9	18.9
	5.984	114.3				
	5.984	114.3				
*N*-C16-l-serine	4.964	145.9	0.5844	58.2	37.4	19.7
	7.542	121.5				
	8.839	92.6				
*N*-C16-dl-serine-ME	4.910	134.5	0.3389	44.8	28.1	19.9
	5.911	120.8				
	6.657	104.7				
*N*-C16-l-serine-ME	4.892	134.4	0.3416	45.4	27.7	19.5
	5.850	121.4				
	6.640	104.2				
*N*-C18-dl-threonine-ME	4.937	137.8	0.3835	49.8	31.6	20.4
	6.864	111.1				
	6.864	111.1				
*N*-C18-l-threonine-ME	4.916	138.2	0.3957	49.6	31.5	20.4
	6.729	114.3				
	7.040	107.5				

a Bottom: Corresponding lattice parameters of racemic and enantiomeric *N*-C16-serine, *N*-C16-serine-ME, and *N*-C18-threonine-ME monolayers. *a*, *b*, *c*, and α, β, γ are the lattice parameters of the unit cell; *t* is the polar tilt angle; *A*_*xy*_ is the molecular area; *A*_0_ is the cross-sectional area of the alkyl chain; and *d* is the lattice distortion.

A characteristic for all structures is the large tilt angle with respect to the surface normal which decreases only marginally by compression. This is an indication for the large size of the head groups and strong interactions between them. Therefore, the monolayer structure is dominated by the head groups.

As already described for *N*-C16- and *N*-C18-threonine,^22^ two Bragg peaks are observed for the racemates, indicating a next-nearest neighbor (NNN) tilted orthorhombic structure. The appearance of orthorhombic structures in racemic monolayers is a clear evidence of compound formation with a congruent transition point due to dominating heterochiral interactions. In 1:1 mixtures of enantiomers, two scenarios can be distinguished: (i) homochirality with favored interaction between the same enantiomers (*E*_d–d_ and *E*_l–l_ > *E*_d–l_) leading to enantiomer separation and (ii) heterochirality with favored interaction between the different enantiomers (*E*_d–d_ and *E*_l–l_ < *E*_d–l_) leading to the formation of a racemic compound.

For the first time, an oblique lattice structure is observed for a 1:1 mixture of amino acid derivatives. The racemic *N*-C16-dl-serine-ME forms an oblique lattice structure, whereas the corresponding *N*-C16-dl-serine forms an orthorhombic lattice with NNN tilted chains. Obviously, the introduction of the methylester reduces in this case the possibility of compound formation due to dominating heterochiral interactions. In the case of *N*-C18-dl-threonine-ME monolayers, for which the racemate forms an orthorhombic lattice as the corresponding *N*-C18-dl-threonine, the presence of two chiral carbon atoms leading to four stereoisomers (2S, 3R; 2R, 3S; 2S, 3S; 2R, 3R) might reduce the effect of the methylester. The corresponding pair potential profile (EPP) conclusions are discussed in detail in ref (25). Based on the cross-sectional areas, the orthorhombic phase of the racemate of *N*-C16-dl-serine with an extremely small *A*_0_ value can be named L_2_′^37^ whereas the orthorhombic phase of the racemates of *N*-C18-dl-threonine-ME and *N*-C18-dl-threonine is an Ov phase.^37^

**Figure 6:**
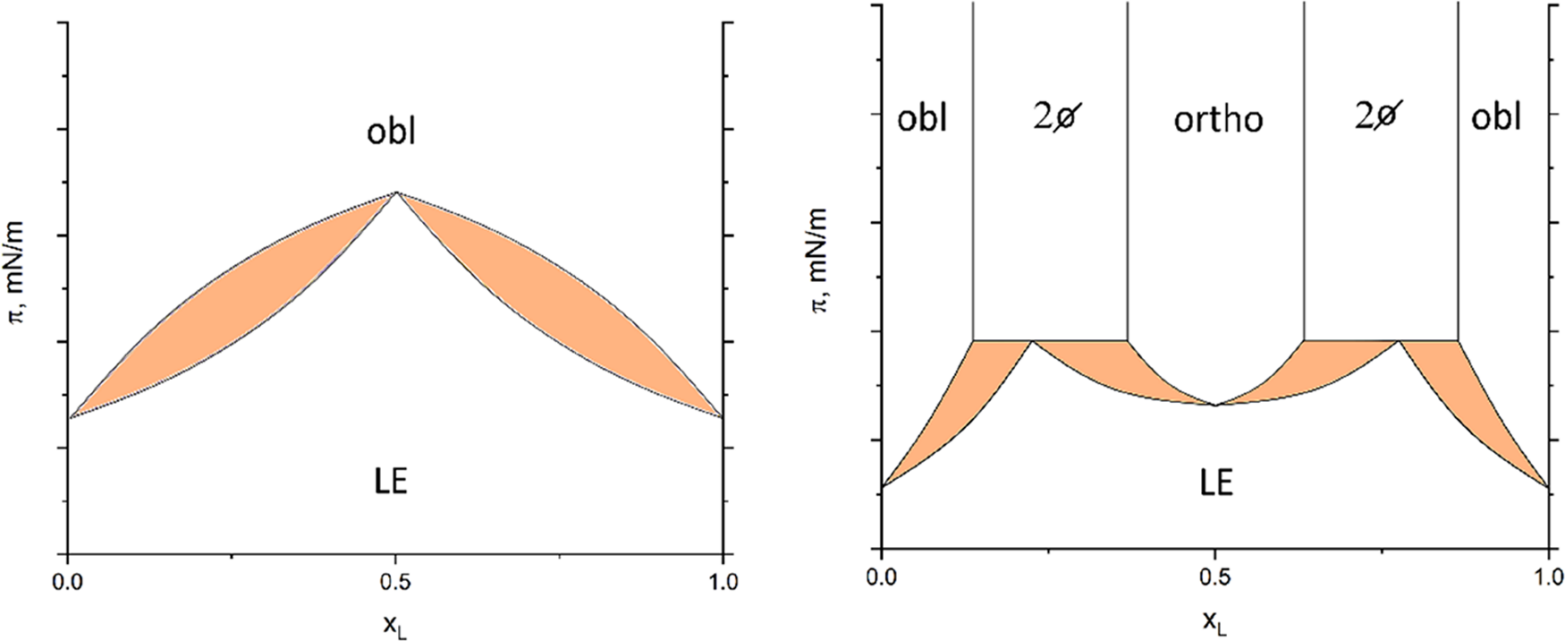
Schematic phase diagrams (lateral pressure π vs mole fraction of the l-enantiomer x_L_) of mixtures of enantiomers. Left: Dominating homochiral interactions lead to an azeotropic point and complete miscibility in both LE and LC phases. The LC phase has an oblique lattice structure. Right: Dominating heterochiral interactions lead to compound formation. Complete miscibility is observed only in the LE phase. The LC phase of the enantiomers (obl) has an oblique lattice structure whereas the LC phase of the racemate (ortho) has an orthorhombic structure (Ov or L_2_′). Miscibility gaps (2Ø) are present between the different LC phases. The colored areas indicate coexistence between LE and LC.

It is interesting to note that the transition pressure of the first-order LE/LC transition is always higher for the 1:1 mixtures compared to the pure enantiomers, even in the case of *N*-C16-dl-serine-ME for which an oblique phase structure has been observed. In this case, the phase diagram does exhibit a rather homogeneous distribution of enantiomers without distinct favored interactions between the different enantiomers (Figure 6, left). Complete miscibility is observed in both the LE and LC phases but with a maximum in the transition pressure at the 1:1 mixture similar to an azeotrope. In contrast, the phase diagram of two enantiomers forming a “congruent melting” (direct change from LE to LC of the same composition) compound can be regarded as two simple eutectic systems (Figure 6, right) combined by a common *y*-axis (lateral pressure). The transition pressure of the compound presents a maximum.

**Figure 7:**
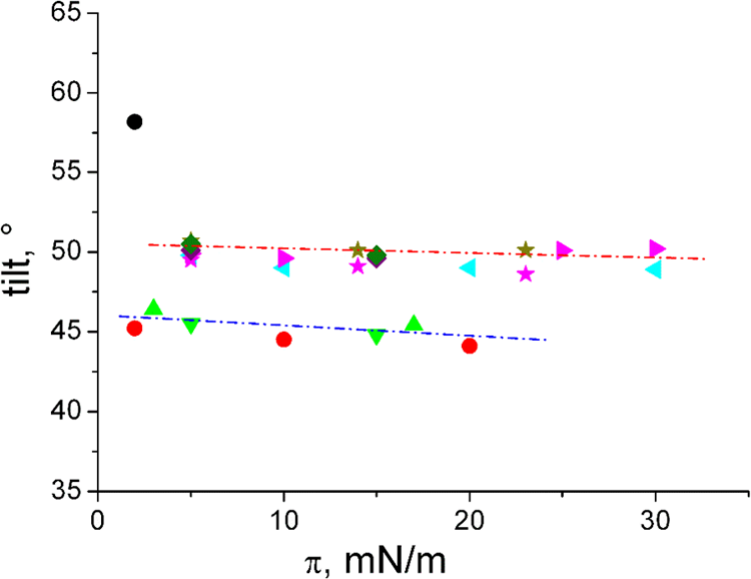
Alkyl chain tilt angle of *N*-C16-dl-serine (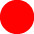), *N*-C16-l-serine (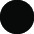), *N*-C16-dl-serine-ME (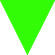), *N*-C16-l-serine-ME (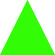), *N*-C18-dl-threonine (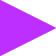), *N*-C18-l-threonine (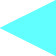), *N*-C18-dl-threonine-ME (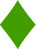), *N*-C18-l-threonine-ME (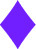), *N*-C18-dl-*allo*-threonine (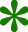), and *N*-C18-l-*allo*-threonine (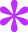) vs lateral pressure π.

The transition pressure into a nontilted phase cannot be calculated by the usually applied extrapolation toward 1/cos(*t*) = 1.^33^ Because of the independence of the tilt angle from the lateral pressure (Figure 7), such an extrapolation does not lead to any reasonable values. A strong hydrogen bonding network between the head groups can be most reasonably assumed (the lattice distortion *d* is also large and almost constant) even if the cross-sectional areas in some cases are typical for rotator phases.^37^

The chain cross-sectional areas *A*_0_ of *N*-C18-threonine, *N*-C18-*allo*-threonine, and *N*-C18-threonine-ME are very similar and typical for a rotator phase (>20 Å^2^), indicating free rotation of the alkyl chains. The tilt angle is ∼50°, also very similar for all compounds (the red line in Figure 7 is only for guiding the eye). Obviously, the packing and area requirement of the molecules is not influenced by the introduction of the methylester. The same is valid for *N*-C16-serine and *N*-C16-serine-ME (blue line in Figure 7) with a tilt angle around 45°. The only exception is the enantiomeric *N*-C16-serine with an extremely large tilt angle of 58° (one of the largest values ever observed in monolayers) but a similar cross-sectional area. The racemic *N*-C16-serine exhibits the tightest packing with a cross-sectional area ∼19 Å^2^.

The molecular packing was calculated by a Monte Carlo algorithm using the program Hardpack^35^ that tries to find the global minimum of the energy of the two-dimensional packing arrangements of molecules. The energy is calculated from van der Waals and electrostatic interactions between different molecules and rotatable groups. The bond length and bond angles of the molecules are fixed. Selected groups are allowed to rotate. Theoretical point charges at the atom positions are used. The molecular structures were optimized by quantum chemical calculations, as presented in Experimental Section.^34^ For each of the six packing types, a few hundred molecular packing arrangements with varying parameters, including six to seven internal rotations, were computed. The cell constants were held fixed at the experimental values. The theoretical calculations produce information about the possible head group conformations. They also find the tilt of the alkyl chain, which can be compared to the experimental values. Table S3 shows the results for all packing arrangements calculated by Hardpack. The theoretically calculated tilt angles of the alkyl chains are slightly larger than the corresponding experimental values. Therefore, the cross-sectional area of the chains is smaller compared to the experimental values. Such small values for amphiphiles in monolayers at the air/water interface may be caused by the negligence of thermal motions in the packing calculations. Thermal motion leads to larger distances between the alkyl chains and hence to larger *A*_0_ values, as experimentally observed.

**Figure 8:**
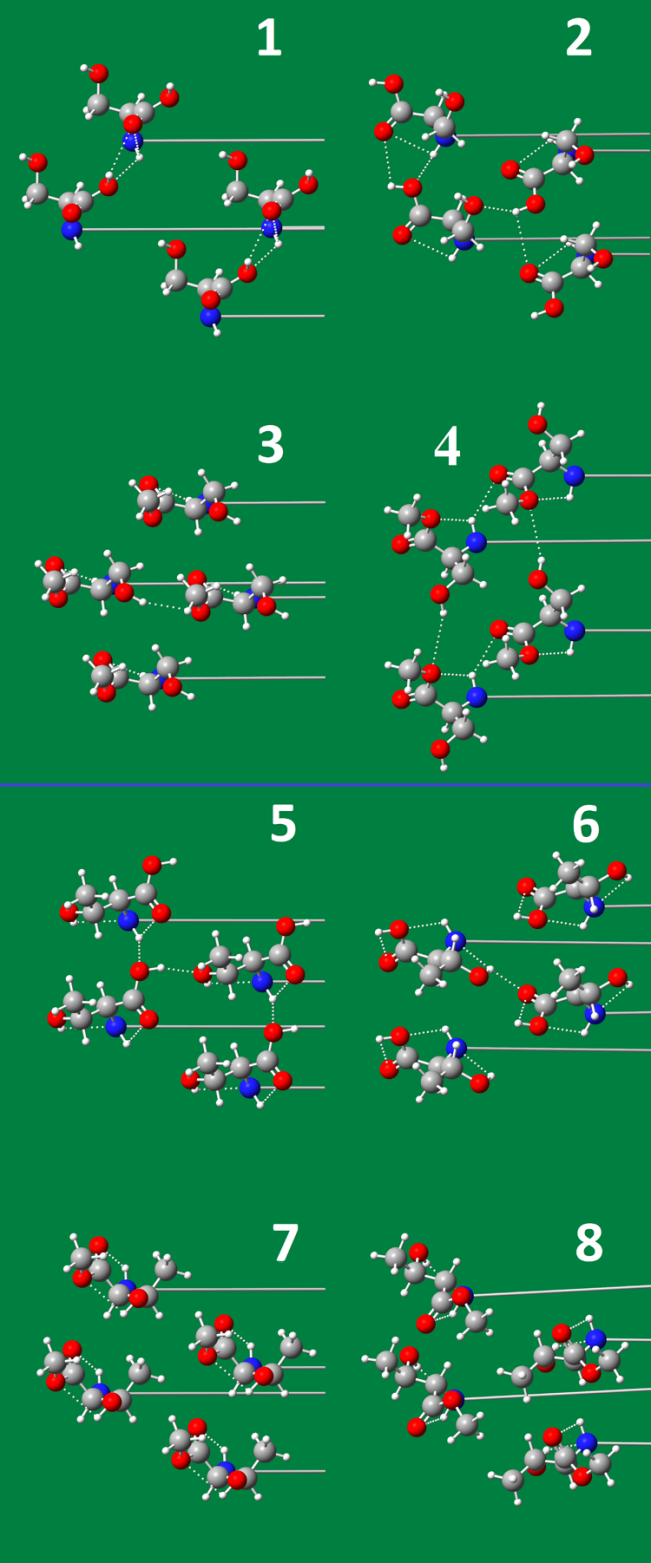
Top: Head group packing arrangements of *N*-C16-serine and *N*-C16-serine-ME, viewed from the water side of the monolayer surface. The hydrogen bonds are shown as dotted lines and the chain directions as long horizontal lines. (1) *N*-C16-l-serine, (2) *N*-C16-dl-serine, (3) *N*-C16-l-serine-ME, and (4) *N*-C16-dl-serine-ME. Bottom: Head group packing arrangements of *N*-C16-threonine and *N*-C16-threonine-ME, viewed from the water side of the monolayer surface. The hydrogen bonds are shown as dotted lines and the chain directions as long horizontal lines. (5) *N*-C16-l-threonine, (6) *N*-C16-dl-threonine, (7) *N*-C16-l-threonine-ME, and (8) *N*-C16-dl-threonine-ME.

Figure 8 shows the packing arrangements of the head groups of all investigated amphiphiles quantum chemically calculated. The head group packing arrangements viewed from the water side of the monolayer surface show the hydrogen bonds as dotted lines and the chain directions as long horizontal lines.

The head group packing arrangements of all presented compounds show that a complex system of inter- and intramolecular hydrogen bonds is formed, affected by the chemical structure of the head groups. In previous papers, we theoretically studied the effect of chiral interaction on the morphology of condensed-phase domains in monolayers of amino acid amphiphiles.^24,25^ Effective Pair Potential studies have shown that the chiral interaction favors a mutual azimuthal orientation between the molecules in the unit cell for all amino acid amphiphiles considered. These results suggest that the hydrogen bond cycles present between the molecules within the domain prevent the tendency of intermolecular twist due to chiral interaction. However, at the interface between the condensed phase and the fluid phase, the hydrogen bonding energy is not as strong as that within the domain and additionally less direction specific. It is concluded that the chiral interaction is dominating at the interface, resulting in the curved shape of the domain.

According to the present Hardpack calculations, the formation of an intermolecular hydrogen bonding network must be the main reason for a stiffening of the monolayer structure. Therefore, the tilt angle of the chains does only marginally decrease with increasing lateral pressure in all of the studied monolayers. But the formation of intramolecular hydrogen bonds is also able to rigidify the head group structure in such a way that it contributes essentially to the packing stability of the monolayers of amino acid-type amphiphiles.

## Conclusions

Thermodynamic analysis of the temperature dependence of pressure–area isotherms of the *N*-alkanoyl-substituted α-amino acids threonine and serine, differing only by one CH_3_ group in the head group, and their corresponding methylesters allows the determination of the characteristic temperatures *T*_0_ and *T*_c_. The introduction of the methyl group in 3-position of the serine (serine to 3-methyl-serine = threonine) shifts these characteristic temperatures by more than 20 K to lower values determined for *N*-C16-dl-serine. The formation of the corresponding methylester decreases these temperatures by 15 K for the serine with the shorter (C16) chain and only by ∼5 K for the threonine with the longer chain (C18). Obviously, the stronger van der Waals interactions of the longer chain decrease the influence of the methylester. The influence of stereochemistry is the same for all investigated samples. The phase-transition pressure from the disordered LE to an ordered LC phase is at any fixed temperature higher for the racemic mixture compared to the pure enantiomers. This observation is even independent of the structure formed by the racemic mixtures. All racemates, except *N*-C16-dl-serine-ME, form orthorhombic lattices whereas all enantiomers form oblique lattices. *N*-C16-dl-serine-ME is the first exception found for *N*-alkanoyl-substituted α-amino acids. Since the racemate of *N*-C16-serine-ME has also a phase-transition pressure above that of the corresponding enantiomers, the tendency of preferred heterochiral interactions in racemic mixtures is present but not strong enough for compound formation. Instead, a phase diagram with an azeotropic point has to be assumed.

Quantum chemical optimizations of the molecular structures and subsequent packing calculations were performed to theoretically predict the tilt angles and cross-sectional areas of the alkyl chains. The obtained results with the used quantum chemical procedure are in reasonable agreement with the experimental GIXD data.

## Supporting Information Available

Temperature dependence of the main phase-transition pressure π_t_ and entropy change Δ*S* of enantiomeric and racemic N-C16-serine-ME (Figure S1), compression and expansion π–A curves to show the overcompression (Figure S2), temperature dependence of π_t_ and Δ*S* of enantiomeric and racemic *N*-C18-threonine-ME (Figure S3), Bragg peak and rod positions and the corresponding lattice parameters of all compounds measured by GIXD (Tables S1–S3), and theoretical parameters (obtained by Hardpack) (Table S4) (PDF)
