# Supplementary material for: Influence of Stereochemistry on the Monolayer Characteristics of N-alkanoyl-Substituted Threonine and Serine Amphiphiles at the Air–Water Interface
Source: Langmuir. 2021 Jul 21;37(30):9069–77. doi: 10.1021/acs.langmuir.1c01108 (PMC8397399; doi:10.1021/acs.langmuir.1c01108)
Supplement: Supplementary file 1 — la1c01108_si_001.pdf [file la1c01108_si_001.pdf]

Supporting Information

Influence of stereochemistry on the monolayer characteristics of N-alkanoyl substituted threonine and serine amphiphiles at the air-water interface

G. Brezesinski[1], F. Strati[1], R. Rudert[2], D. Vollhardt[3]*

[1]Institute for Applied Dermatopharmacy, Martin Luther University Halle-Wittenberg, Weinbergweg 23, D-06120 Halle, Germany
[2]Section of Chemical Information Systems, University of Ulm, D-89081 Ulm, Germany
[3]Max-Planck Institute for Polymer Research, Ackermannweg 10, D-55128 Mainz, Germany

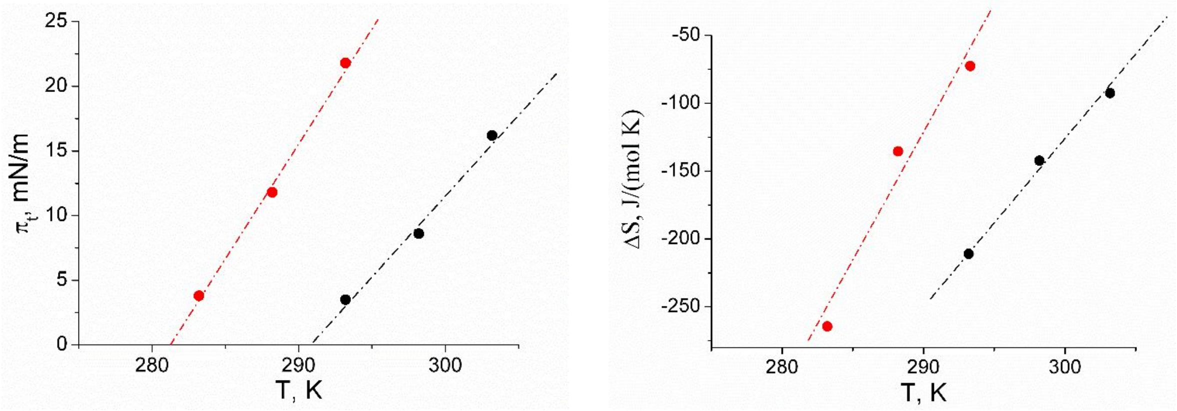

Figure S1. *Left*: Temperature dependence of the main phase-transition pressure π_t at the LE/LC phase transition of enantiomeric (black) and racemic (red) *N*-C16-serine-ME monolayers spread on pH 3 water. *Right*: Temperature dependence of the entropy change ΔS at the LE/LC phase transition of enantiomeric (black) and racemic (red) *N*-C16-serine-ME monolayers spread on pH 3 water.

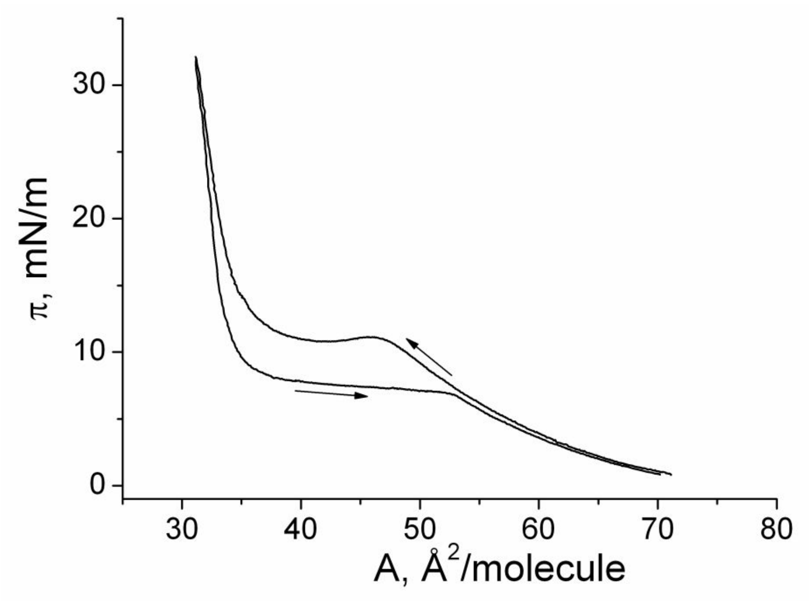

Figure S2. Comparison of compression and expansion π-A curves of *N*-C18-*DL*-threonine-ME at 16 °C. Overcompression is observed in the compression isotherm.

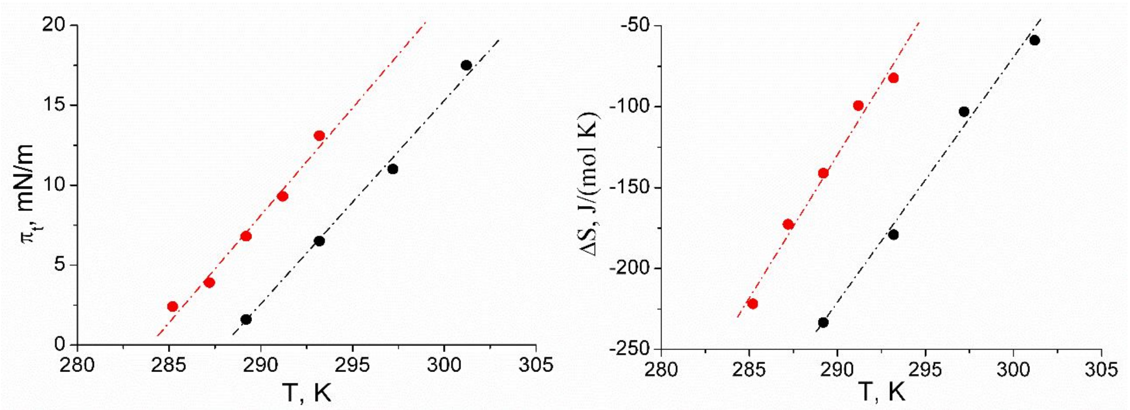

Figure S3. *Left*: Temperature dependence of the main phase-transition pressure π_t at the LE/LC phase transition of enantiomeric (black) and racemic (red) *N*-C18-threonine-ME monolayers spread on water. *Right*: Temperature dependence of the entropy change ΔS at the LE/LC phase transition of enantiomeric (black) and racemic (red) *N*-C18-threonine-ME monolayers spread on water.

Table S1. *Top*: Bragg peak and rod positions and the corresponding Full-Widths at Half-Maximum of enatiomeric and racemic *N*-C18-threonine-ME monolayers at different lateral pressures π (indicated) measured on pH 3 subphase at 10 °C.
Bottom: Corresponding lattice parameters of racemic and enatiomeric *N*-C18-threonine-ME monolayers.

C18-L-threonine-ME

π<br>mN/m	Q_{xy}<br>$Å^{-1}$	Q_z<br>$Å^{-1}$	Q_{xy}<br>$Å^{-1}$	Q_z<br>$Å^{-1}$	Q_{xy}<br>$Å^{-1}$	Q_z<br>$Å^{-1}$
5	0.970<br>0.029	1.16<br>0.28	1.337<br>0.012	0.47<br>0.26	1.396<br>0.011	0.69<br>0.28
15	0.979<br>0.042	1.15<br>0.28	1.340<br>0.013	0.49<br>0.27	1.402<br>0.017	0.66<br>0.28

π<br>mN/m	a, b, c<br>Å	α, β, γ<br>°	d	t<br>°	A_{xy}<br>$Å^2$	A_0<br>$Å^2$
5	4.927<br>6.792<br>7.091	138.5<br>114.0<br>107.5	0.40098	50.1	31.9	20.5
15	4.916<br>6.729<br>7.040	138.2<br>114.3<br>107.5	0.39572	49.6	31.5	20.4

C18-DL-threonine-ME

π mN/m	Q_{xy} $Å^{-1}$	Q_z $Å^{-1}$	Q_{xy} $Å^{-1}$	Q_z $Å^{-1}$	Q_{xy} $Å^{-1}$	Q_z $Å^{-1}$
5	0.973<br>0.095	1.18<br>0.28	1.363<br>0.013	0.59<br>0.28		
15	0.981<br>0.045	1.16<br>0.27	1.364<br>0.014	0.58<br>0.28		

π mN/m	a, b, c Å	α, β, γ °	d	t °	A_{xy} $Å^2$	A_0 $Å^2$
5	4.935<br>6.913<br>6.913	138.2<br>110.9<br>110.9	0.39081	50.5	31.9	20.3
15	4.937<br>6.864<br>6.864	137.8<br>111.1<br>111.1	0.38354	49.8	31.6	20.4

Table S2. *Top*: Bragg peak and rod positions and the corresponding Full-Widths at Half-Maximum of enatiomeric and racemic *N*-C16-serine-ME monolayers at different lateral pressures π (indicated) measured on water at 10 °C.
Bottom: Corresponding lattice parameters of racemic and enatiomeric *N*-C16-serine-ME monolayers.

C16-L-serine-ME

π mN/m	Q_{xy} $Å^{-1}$	Q_z $Å^{-1}$	Q_{xy} $Å^{-1}$	Q_z $Å^{-1}$	Q_{xy} $Å^{-1}$	Q_z $Å^{-1}$
3	1.107<br>0.027	1.16<br>0.30	1.324<br>0.008	0.42<br>0.28	1.501<br>0.013	0.74<br>0.30
17	1.108<br>0.034	1.12<br>0.30	1.325<br>0.008	0.42<br>0.28	1.504<br>0.013	0.70<br>0.29

π mN/m	a, b, c Å	α, β, γ °	d	t °	A_{xy} $Å^2$	A_0 $Å^2$
3	4.898<br>5.858<br>6.641	134.4<br>121.3<br>104.3	0.34025	46.4	27.8	19.2
17	4.892<br>5.850<br>6.640	134.4<br>121.4<br>104.2	0.34158	45.4	27.7	19.5

C16-DL-serine-ME

π mN/m	Q_{xy} $Å^{-1}$	Q_z $Å^{-1}$	Q_{xy} $Å^{-1}$	Q_z $Å^{-1}$	Q_{xy} $Å^{-1}$	Q_z $Å^{-1}$
5	1.094	1.11	1.322	0.41	1.488	0.70

	0.036	0.3	0.009	0.3	0.017	0.3
15	1.099<br>0.040	1.109<br>0.3	1.323<br>0.009	0.41<br>0.28	1.490<br>0.017	0.68<br>0.29

π<br>mN/m	a, b, c<br>Å	α, β, γ<br>°	d	t<br>°	A_{xy}<br>$Å^2$	A_0<br>$Å^2$
5	4.912<br>5.936<br>6.681	134.6<br>120.7<br>104.6	0.34196	45.5	28.2	19.8
15	4.910<br>5.911<br>6.657	134.5<br>120.8<br>104.7	0.33889	44.8	28.1	19.9

Table S3. *Top*: Bragg peak and rod positions and the corresponding Full-Widths at Half-Maximum of enatiomeric and racemic *N*-C16-serine monolayers at different lateral pressures π (indicated) measured on pH 2.5 subphase at 10 °C.
Bottom: Corresponding lattice parameters of racemic and enatiomeric *N*-C16-serine monolayers.

C16-L-serine

π<br>mN/m	Q_{xy}<br>$Å^{-1}$	Q_z<br>$Å^{-1}$	Q_{xy}<br>$Å^{-1}$	Q_z<br>$Å^{-1}$	Q_{xy}<br>$Å^{-1}$	Q_z<br>$Å^{-1}$
2	0.834<br>0.044	1.33<br>0.30	1.267<br>0.009	0.41<br>0.30	1.485<br>0.020	0.92<br>0.30

π<br>mN/m	a, b, c<br>Å	α, β, γ<br>°	d	t<br>°	A_{xy}<br>$Å^2$	A_0<br>$Å^2$
2	4.964<br>7.542<br>8.839	145.9<br>121.5<br>92.6	0.58435	58.2	37.4	19.7

C16-DL-serine

π<br>mN/m	Q_{xy}<br>$Å^{-1}$	Q_z<br>$Å^{-1}$	Q_{xy}<br>$Å^{-1}$	Q_z<br>$Å^{-1}$	Q_{xy}<br>$Å^{-1}$	Q_z<br>$Å^{-1}$
2	1.152<br>0.024	1.16<br>0.31	1.400<br>0.009	0.58<br>0.29		
10	1.162<br>0.033	1.14<br>0.31	1.402<br>0.011	0.57<br>0.31		
20	1.157<br>0.037	1.12<br>0.30	1.403<br>0.013	0.56<br>0.31		

π<br>mN/m	a, b, c<br>Å	α, β, γ<br>°	d	t<br>°	A_{xy}<br>$Å^2$	A_0<br>$Å^2$
2	4.924<br>5.984	131.4<br>114.3	0.24124	45.2	26.9	18.9

	5.984	114.3				
10	4.924<br>5.941<br>5.941	131.0<br>114.5<br>114.5	0.23303	44.5	26.6	19.0
20	4.916<br>5.961<br>5.961	131.3<br>114.4<br>114.4	0.23875	44.1	26.7	19.2

Table S4. Theoretical parameters (Hardpack)

	a [Å]	b [Å]	c [Å]	α [°]	β [°]	γ [°]	**t [°]**	*t [°]*	A_{xy} *[Å²]*	**A_0 [Å²]**	A_0 *[Å²]*
N-C16-*L*-serine	*4.964*	*7.542*	*8.839*	*145.9*	*121.5*	*92.6*	**60.6**	*58.2*	*37.4*	**18.3**	*19.7*
N-C16-*DL*-serine	*4.924*	*5.984*	*5.984*	*131.4*	*114.3*	*114.3*	**47.6**	*45.2*	*26.9*	**18.1**	*18.9*
N-C16-*L*-serine-ME	*4.898*	*5.858*	*6.641*	*134.4*	*121.3*	*104.3*	**50.7**	*46.4*	*27.8*	**17.6**	*19.2*
N-C16-*L*-threonine*	*4.922*	*6,714*	*7.035*	*138.1*	*114.4*	*107.4*	**56.0**	*48.8*	*31.5*	**17.7**	*20.7*
N-C16-*DL*-threonine*	*4.923*	*6.772*	*6.772*	*137.4*	*111.3*	*111.3*	**54.7**	*50.0*	*31.1*	**18.1**	*20.0*
N-C16-*L*-threonine-ME	*4.927*	*6.792*	*7.091*	*138.5*	*114.0*	*107.5*	**54.7**	*50.1*	*31.9*	**18.4**	*20.5*
N-C16-*DL*-threonine-ME	*4.935*	*6.913*	*6.913*	*138.2*	*110.9*	*110.9*	**53.0**	*50.5*	*31.9*	**19.2**	*20.3*

Italic numbers are from GIXD measurements and were used for the theoretical calculations.
Bold numbers are data calculated by Hardpack.
*) experimental values from *N*-C16-threonine [1]

(1) Vollhardt, D.; Stefaniu, C.; Brezesinski, G. Special features of monolayer characteristics of N-alkanoyl substituted threonine amphiphiles. *Phys. Chem. Chem. Phys.* **2019**, *21*, 96-103.
